# Corticosteroid injection for carpal tunnel syndrome: A meta-analysis comparing ultrasound guided approach with landmark approach

**DOI:** 10.12669/pjms.40.4.8749

**Published:** 2024

**Authors:** Anqing Jiang, Yushun Qian, Jun Yan, Shouchun Zhang, Siqiang Zhu

**Affiliations:** 1 Anqing Jiang, Department of Orthopedics, The Second People’s Hospital of Xiangcheng District, Suzhou City, Suzhou 215131, Jiangsu Province, P.R. China; 2 Yushun Qian, Department of Orthopedics, The Second People’s Hospital of Xiangcheng District, Suzhou City, Suzhou 215131, Jiangsu Province, P.R. China; 3 Jun Yan, Department of Orthopedics, The Second People’s Hospital of Xiangcheng District, Suzhou City, Suzhou 215131, Jiangsu Province, P.R. China; 4 Shouchun Zhang, Department of Orthopedics, The Second People’s Hospital of Xiangcheng District, Suzhou City, Suzhou 215131, Jiangsu Province, P.R. China; 5 Siqiang Zhu, Department of Orthopedics, The Second People’s Hospital of Xiangcheng District, Suzhou City, Suzhou 215131, Jiangsu Province, P.R. China

**Keywords:** Carpal tunnel syndrome, Median nerve, Steroid injection, Ultrasound guided approach, Landmark guided approach, Meta-analysis

## Abstract

**Objective:**

To assess and compare the clinical and functional outcomes of corticosteroid injections in patients with carpal tunnel syndrome, focusing on two different approaches: ultrasound-guided and landmark-guided.

**Methods:**

A systematic search was conducted in PubMed, Scopus and Embase databases for relevant studies published prior to 30^th^ April 2023. Studies that were either randomized controlled trials or had a cohort design were included. The review assessed symptom severity, functional status, electrodiagnostic parameters, complications, need for surgical intervention, visual analogue score, and grip strength. Pooled effect sizes were reported as relative risk (RR) or weighted mean difference (WMD).

**Results:**

A total of 8 articles were included. Compared to those that received steroid injection using landmark approach, those with ultrasound guided approach had lower symptom severity scale (SSS) score on Boston Carpal Tunnel Questionnaire (BCTQ) [WMD -0.50, 95% CI: -0.94, -0.07; I^2^=78.0%, N=7], lower risk of “any complications” [RR 0.58, 95% CI: 0.36, 0.93; I^2^= 22.9%, N=3] and lower risk of need for surgical intervention [RR 0.55, 95% CI: 0.34, 0.89; I^2^= 3.0%, N=2]. All other parameters were similar in the two groups i.e., functional status scale (FSS) score, visual analogue score (VAS) and grip strength. The electrophysiological findings were similar in the two groups.

**Conclusion:**

Findings suggest that ultrasound guided approach may be better than landmark guided approach especially in terms of alleviation of symptoms, reducing the risk of complications and need for surgical intervention. However, larger trials with long term follow up may provide conclusive evidence.

## INTRODUCTION

Carpal tunnel syndrome is common clinical condition, often presenting as pain and paraesthesia in the area supplied by median nerve.^1^,^2^ It primarily arises from the compression of the median nerve within the carpal tunnel, located in the wrist.^1^,^2^ Non-surgical interventions are typically employed to manage mild to moderate cases of carpal tunnel syndrome. These interventions include the use of anti-inflammatory medications, local insulin injections, platelet-rich plasma therapy, and corticosteroids.^3^–^6^ Clinicians conventionally use anatomical landmarks to identify appropriate injection sites and avoid injury to the median nerve and surrounding neurovascular bundle when administering these interventions. Recently, ultrasound has increased in popularity for this purpose. Ultrasound can help visualize structures within the carpal tunnel in real time, thereby diminishing the possibility of nerve, tendon, and vasculature iatrogenic injury.^7^,^8^

There is a lack of consensus regarding whether ultrasound-based or landmark-based approaches are superior for carpal tunnel syndrome interventions. A previous meta-analysis, consisting of three randomized controlled trials, showed that ultrasound-guided steroid injections improved symptom severity more than landmark-guided injections, but had no significant impact on functional severity.^9^ Similar studies in patients with shoulder pain also showed that ultrasound-guided interventions reduced pain but did not affect function.^10^ As more information has been made available since that initial meta-analysis, there is a current need to re-evaluate the available evidence. With the increasing recognition of the possible role that trained and motivated nursing personnel can perform in the management of carpal tunnel syndrome, it becomes extremely crucial that these specialized cadre of clinical team remain updated with the recent evidence.^11^

## METHODS

### Search strategy and selection of studies

We systematically searched PubMed, Scopus and Embase databases for English language papers published prior to 30^th^ April 2023.The search strategy was: (ultrasound guided OR USG guided OR landmark guided OR blind) AND (steroid injection OR triamcinolone OR betamethasone OR methylprednisolone) AND (carpal tunnel syndrome). This search sought to identify studies on carpal tunnel syndrome patients comparing outcomes when ultrasound-guided and landmark-guided/blinded steroid injection was employed. Primary outcomes of interest were functional status, symptom severity, and electrophysiological parameters. Secondary outcomes included were visual analog scale system scores, grip strength, complication rates, and need for surgical intervention. PRISMA guidelines^12^ were followed during the conduct of this meta-analysis. The protocol was registered at PROSPERO (https://www.crd.york.ac.uk/prospero/, No. CRD42023434696).

Two subject experts reviewed the studies identified through the search strategy. The initial screening phase consisted of reviewing the titles and abstracts, with eligible studies advancing to full-text review. Any disagreements between the reviewers were resolved through discussion. To be included, studies must have been either a clinical trial or had a cohort design (either prospective or retrospective). Studies must have investigated patients with carpal tunnel syndrome, with the intervention used must have been steroid injection. Finally, studies must have compared outcomes of interest when steroid injection was ultrasound-guided versus landmark-guided/blinded.

### Data Extraction, quality assessment and statistical analysis

A standardized sheet was used to extract data relevant to this study. Study methodologies were assessed using the Cochrane assessment tool for randomized controlled trials or the Newcastle-Ottawa Quality Assessment Scale (NOS) for observational studies.^13^,^14^ Meta-analysis was carried out using STATA version 16.0. For categorical outcomes, the effect sizes were presented as pooled relative risk (RR). Continuous outcomes, on the other hand, were reported as weighted mean differences (WMD). All the effect sizes were reported along with 95% confidence intervals. Random effects model was employed in case of significant heterogeneity, indicated by an I^2^ value of more than 40%.^15^ To determine statistical significance, a threshold of p-values <0.05 was employed. Publication bias was assessed using Egger’s test.

## RESULTS

**Fig.1:**
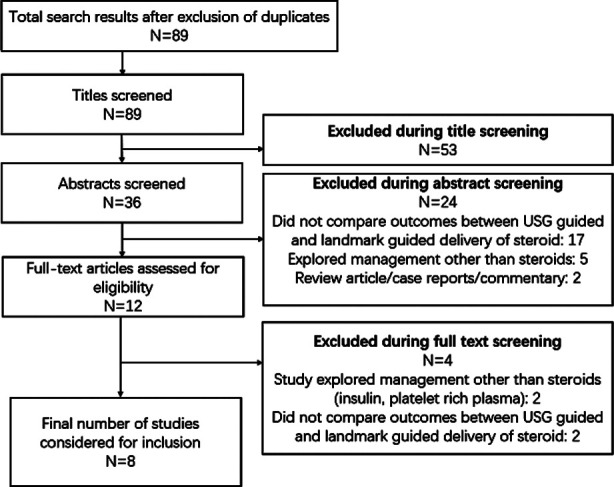
Study screening process.

Database searching returned 131 studies of interest (Fig.1). Title review eliminated 83 studies, while abstract review eliminated a further 32. This left 16 studies for full-text review, after which eight articles met inclusion criteria and were included in the meta-analysis (Table-I).^16^-^23^ Two studies each was conducted in Turkey, Iran, and South Korea, with the USA and Taiwan hosting one study each. All studies except one (a retrospective examination)^21^ were randomized controlled trials. Four studies used triamcinolone as the injected steroid while two studies each used betamethasone and methylprednisolone (Table-I). All studies but one (12 months) assessed outcomes within six months of intervention. All the studies reported random sequence generation and allocation concealment. All studies, except for one,^22^ reported blinding of the outcome assessment team. The lone observational study was of satisfactory quality, obtaining a score of seven out of the maximum attainable score of nine on NOS.

**Table-I:** Study characteristics and extracted data

Author (year of publication)	Study design	Country	Participant characteristics	Sample size
Chen PC et al (2018)^15^	Double blind randomized controlled trial	Taiwan	Patients with carpal tunnel syndrome; mean age of around 50 years and symptom duration of around 65 months; mean body mass index of around 25 kg/m^2^ Patients received betamethasone dipropionate 5 mg and betamethasone disodium phosphate 2 mg.	22 with sonography approach and 17 with landmark guided approach
Eslamian F et al (2017)^16^	Randomized controlled trial	Iran	Patients with carpal tunnel syndrome; mean age of around 50 yrs; >80% females; around one-fifth had a history of diabetes mellitus Patients received 40 mg of methylprednisolone	27 with sonography approach and 20 with landmark guided approach
Roh YH et al (2019)^17^	Randomized controlled trial	South Korea	Patients with carpal tunnel syndrome; mean age of around 54 yrs; Majority females (78%); median duration of symptoms was 15 months; in 40% patients dominant hand was involved. Patients received single 2-mL injection that contained 1 mL of lidocaine (10 mg/mL) and 1 mL of triamcinolone acetonide (20 mg/mL).	51 with sonography approach and 51 with landmark guided approach
Ustun N et al (2013)^18^	Randomized controlled trial	Turkey	Patients with carpal tunnel syndrome; mean age of around 45 yrs; Majority females; duration of symptoms ranging between 10-15 months. Patients received 40 mg of methylprednisolone	23 with sonography approach and 23 with landmark guided approach
Rayegami SM et al (2019)^19^	Randomized controlled trial	Iran	Patients with carpal tunnel syndrome; mean age of around 54 yrs; Majority females; Majority had left sided hand dominance. Patients received a mixture of 1 mL of triamcinolone 40 mg and 1 mL of lidocaine (2%)	26 with sonography approach and 23 with landmark guided approach
Evers S et al (2017)^20^	Retrospective database study	USA	Patients with carpal tunnel syndrome; mean age of around 50 yrs; Majority females (around 70%). Patients received triamcinolone injections	87 with sonography approach and 234 with landmark guided approach
Lee JY et al (2014)^21^	Randomized controlled trial	South Korea	Patients with mild to moderate idiopathic carpal tunnel syndrome; mean age of 50 yrs; majority were female; mean duration of symptoms was around 9 months. Patients received 1 ml of 40 mg/mL triamcinolone and 1mL of 1% lidocaine	15 with sonography approach and 15 with landmark guided approach
Karaahmet OZ et al (2017)^22^	Prospective, randomized clinical trial	Turkey	Patients with carpal tunnel syndrome; mean age of around 61 years and most of them were females and mostly the dominant hand was right hand 1 mL of betamethasone sodium phosphate (2.63 mg)/ betamethasone dipropionate (6.43 mg) was injected	15 with sonography approach and 16 with landmark guided approach

### Physical findings

**Fig.2:**
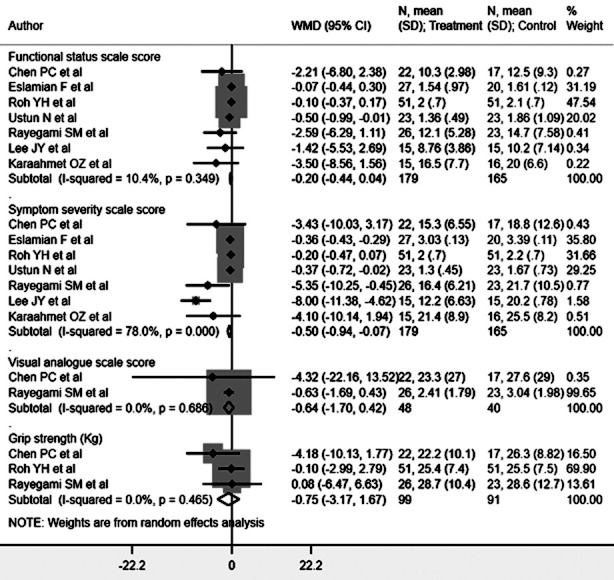
Functional status, symptom severity, visual analogue score, and grip strength for carpal tunnel syndrome patients receiving ultrasound guided and landmark guided steroid injection.

**Fig.3:**
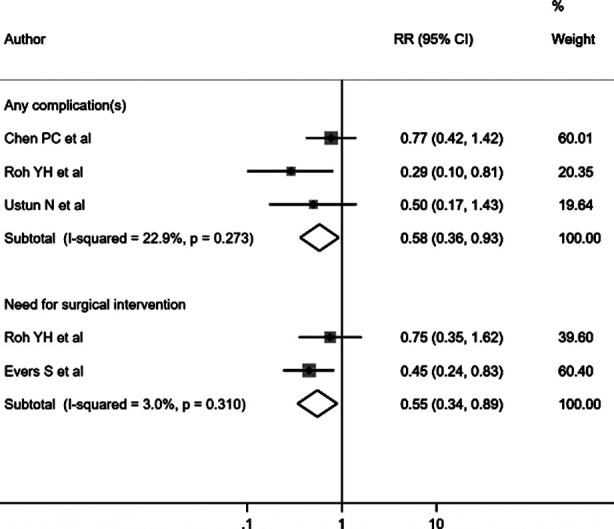
Risk of “any complication(s)” and “need for surgical intervention” for carpal tunnel syndrome patients receiving ultrasound guided and landmark guided steroid injection.

Carpal tunnel syndrome patients receiving ultrasound-guided steroid injections showed decreased symptom severity scale (SSS) scores, as measured using the Boston Carpal Tunnel Questionnaire (BCTQ) [WMD -0.50, 95% CI: -0.94, -0.07; I^2^=78.0%, N=7] compared to patients receiving landmark-guided steroid injection (Fig.2). No significant differences were noted in functional status scale (FSS) score [WMD -0.20, 95% CI: -0.44, 0.04; I^2^=10.4%, N=7], visual analogue score (VAS) [WMD -0.64, 95% CI: -1.70, 0.42; I^2^=0.0%, N=2], or grip strength [WMD -0.75, 95% CI: -3.17, 1.67; I^2^=0.0%, N=3] (Fig.2). Egger’s test did not indicate publication bias (P=0.17 for SSS score, P=0.23 for FSS score, P=0.51 for VAS, and P=0.19 for grip strength). However, patients receiving ultrasound-guided injections had lower risk for “any complications” [RR 0.58, 95% CI: 0.36, 0.93; I^2^= 22.9%, N=3] and showed a decreased need for surgical intervention [RR 0.55, 95% CI: 0.34, 0.89; I^2^= 3.0%, N=2] (Fig.3). Egger’s test did not indicate publication bias (P=0.87 for risk of complications and P=0.42 for risk of surgical intervention).

Numbness, swelling, pain and weakness were the commonly reported complications in the first week of injection, in the study by Chen et al.^16^ Numbness in the wrist was reported by one subject (N=1/22) in the ultrasound guided group and four subjects (N=4/17) in the landmark-guided group. Four subjects in the USG guided group (N=4/22) and six subjects in the landmark guided group (N=6/17) reported swelling in the wrist. In both the groups, 10 subjects reported pain after injection. While no subject in the USG guided group reported weakness of the wrist, three in the landmark guided group (N=3/17) reported on weakness while flexion and extension of wrist. All the reported symptoms were temporary in nature. Roh et al. reported symptoms related to median nerve irritation (N=1/51 in USG group and N=7/51 in landmark guided group), skin discolouration/subcutaneous fat atrophy (N=1/51 in USG group and N=3/51 in landmark guided group) and symptoms of steroid flare (N=2/51 in USG group and N=3/51 in landmark guided group).^18^ Ustun et al. did not report any major complication in the form of nerve or blood vessel injury. Pain during procedure was reported by eight subjects (N=8/23) in the landmark guided group and four subjects (N=4/23) in the USG guided group.^19^ No serious complications were noted in any of the two groups.

### Electrophysiological findings

**Fig.4:**
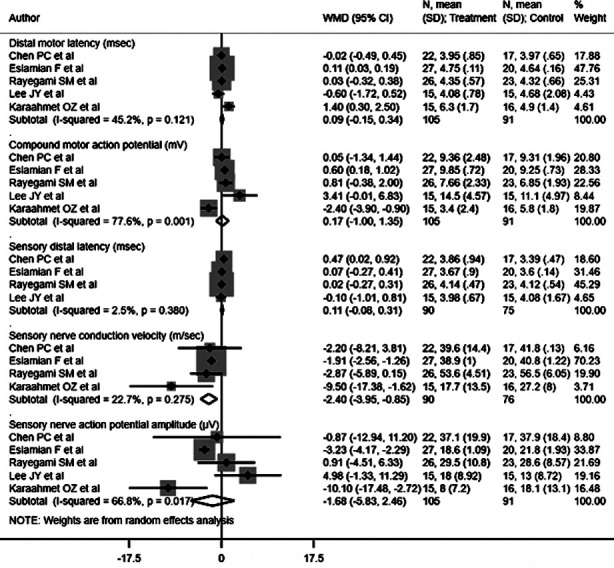
Electrophysiological findings for carpal tunnel syndrome patients receiving ultrasound guided and landmark guided steroid injection.

No statistically significant differences were found between patients receiving ultrasound-guided and landmark-guided injections in terms of distal motor latency (DML) [WMD 0.09, 95% CI: -0.15, 0.34; I^2^=45.2%, N=5], compound motor action potential (CMAP) [WMD 0.17, 95% CI: -1.00, 1.35; I^2^=77.6%, N=5], sensory distal latency (SDL) [WMD 0.11, 95% CI: -0.08, 0.31; I^2^=2.5%, N=4], and sensory nerve action potential amplitude (SNAP) [WMD -1.68, 95% CI: -5.83, 2.46; I^2^=66.8%, N=5] (Fig.4). However, patients receiving ultrasound-guided steroid injections showed decreased sensory nerve conduction velocities (SNCV) [WMD -2.40, 95% CI: -3.95, -0.85; I^2^=22.7%, N=4] (Fig.4). Egger’s test did not indicate publication bias (P=0.36 for DML, P=0.12 for CMAP, P=0.37 for SDL, P=0.54 for SNAP, and P=0.87 for SNCV).

## DISCUSSION

Recent years have seen the wider adoption of ultrasound-based techniques for treating musculoskeletal disorders. The non-invasive nature of ultrasound, combined with its low cost, makes it simple to use for orthopaedic diagnostic evaluation.^24^,^25^ For carpal tunnel syndrome intervention, ultrasound aids injection needle navigation, thereby limiting damage to critical surrounding structures.^7^,^25^ However, the efficacy of ultrasound-guided injections versus traditional landmark-based approaches has not been conclusively established. Our current study showed that patients receiving ultrasound-guided injections presented lower symptom severity and decreased complications risk and need for surgical intervention. These findings are in alignment with other meta-analyses that showed improved symptom severity but no functional severity changes for patients receiving ultrasound-guided injections.^9^,^10^

The 2018 review by Ghazani et al. serves as a foundation, incorporating three randomized controlled trials, but the evolving landscape of research prompts the necessity for an updated meta-analysis.^9^ Recognizing the importance of aligning contemporary clinical practices with the most recent evidence, our meta-analysis expands upon the prior review. Not only do we delve into outcomes previously explored, such as pain scores and grip strength, but we also introduce new dimensions to the analysis. This includes an examination of outcomes like the “risk of complications” and the “need for surgical intervention,” thereby augmenting the comprehensive nature of our study. Our focus extends to providing a detailed exploration of observed complications, adding valuable insights to the existing body of knowledge on the subject. This updated meta-analysis seeks to contribute significantly to informing current clinical practices with a nuanced understanding of the latest evidence.

In particular, our study findings have significant clinical implications and indicate that ultrasound-guided interventions may lead to improved symptom alleviation, potentially reducing the risk of complications associated with injections and minimizing the need for surgical intervention. The shift towards ultrasound guidance indicates a move towards personalized treatment plans, allowing practitioners to tailor interventions based on individual anatomy and severity of symptoms. This approach could enhance the efficiency of healthcare resources, potentially leading to cost savings and optimized resource allocation. The findings also underscore the importance of training healthcare professionals in ultrasound-guided techniques to ensure effective utilization of this technology in clinical practice. Overall, the adoption of ultrasound guidance in managing carpal tunnel syndrome holds promise for advancing patient care, safety, and resource efficiency in the clinical setting.

From the perspective of a nursing personnel, the findings are relevant, given the growing recognition that nursing team may be competent enough to perform standard management techniques for carpal tunnel syndrome. Research findings have indicated that the quality of patient management provided by nursing practitioners in cases of carpal tunnel syndrome is comparable to that of surgeons.^11^,^26^,^27^ It is worth re-iterating that performing ultrasound-guided injections requires extensive training as ultrasound is an operator-dependent instrument. The efficacy of intervention is closely linked to the experience and proficiency of the operator. The operator’s ability to interpret ultrasound images, identify anatomical structures, and guide the needle accurately to the target area is critical for the success of the procedure. For this reason, healthcare professionals, planning to undertake this guided procedure need to undergo specialized training programs to learn the principles of ultrasound imaging and the precise manipulation of the ultrasound probe.

There were differences in the injection site and/or approach method used in the included studies and that should be considered while making interpretations based on the current findings. For instance, in the study by Chen et al and Ustun et al., an out-plane approach was used for the USG guided technique with the transducer placed perpendicular to the median nerve.^16^,^19^ On the other hand, the study by Lee et al. and Karaahmet et al. used an in-plane approach and the needle was started from the ulnar aspect of the transducer while keeping the median nerve in view.^22^,^23^ There were also differences in the experience of the surgeons performing the techniques in the included studies. These might account for the heterogeneity noted for some of the outcomes. This meta-analysis provides updated evidence by pooling the most recent studies on the subject. Low heterogeneity was noted for most of the outcomes of interest, indicating that the included studies adopted similar methodologies.

### Limitations

The eligible studies were few in number with relatively small sample sizes. Performing a subgroup analysis based on the type of corticosteroid utilized would have been beneficial in assessing and comparing complications associated with its use. However, there were three studies that reported complication and all three studies had different type of corticosteroids used. Chen et al used betamethasone dipropionate and betamethasone disodium phosphate; Roh et al. used triamcinolone acetonide and Ustun et al used methylprednisolone.^16^,^18^,^19^ Therefore, conducting a subgroup analysis for complications based on type of corticosteroid had limited benefit.

## CONCLUSION

Our meta-analysis and review suggest that ultrasound-guided steroid injection may be superior to conventional landmark-guided approaches in patients with carpal tunnel syndrome. The benefits of using the ultrasound-guided approach include better alleviation of symptoms, lower risk of complications, and lower risk of surgical intervention. This review also highlights the need for more studies with larger sample sizes for this issue.

Another issue, though not directly related to the findings of this study is the widespread adoption of touch screen devices, along with the ever-growing utilization of computers in various aspects of daily life, that could lead to an increase in the incidence of wrist joint ailments, notably including carpal tunnel syndrome, among the younger population.^28^,^29^ This shift in technology usage patterns has brought about a pressing need for comprehensive research endeavours aimed at understanding and addressing these emerging health concerns. Future studies in this domain must concentrate on elucidating the optimal methods of intervention and therapeutic delivery specifically tailored to the needs of this younger population.

### Authors’ contributions:

**AJ** conceived and designed the study.

**YQ, JY, SZ and SZ** collected the data and performed the analysis.

**AJ** was involved in the writing of the manuscript and is responsible for the integrity of the study.

All authors have read and approved the final manuscript.
